# The testis-specific Cα2 subunit of PKA is kinetically indistinguishable from the common Cα1 subunit of PKA

**DOI:** 10.1186/1471-2091-12-40

**Published:** 2011-08-03

**Authors:** Maike M Vetter, Hans-M Zenn, Eva Méndez, Heidrun van den Boom, Friedrich W Herberg, Bjørn S Skålhegg

**Affiliations:** 1 Department of Biochemistry, University of Kassel, Heinrich-Plett-Str, 40, 34132 Kassel, Germany; 2 Department of Nutrition, Institute of Basic Medical Sciences, University of Oslo, Pb 1046 Blindern, 0316 Oslo Norway; 3 Department of Biochemistry, Institute of Basic Medical Sciences, University of Oslo, Pb 1046 Blindern, 0316 Oslo Norway

**Keywords:** PKA, Catalytic subunit, N-terminal, splice variants

## Abstract

**Background:**

The two variants of the α-form of the catalytic (C) subunit of protein kinase A (PKA), designated Cα1 and Cα2, are encoded by the *PRKACA *gene. Whereas Cα1 is ubiquitous, Cα2 expression is restricted to the sperm cell. Cα1 and Cα2 are encoded with different N-terminal domains. In Cα1 but not Cα2 the N-terminal end introduces three sites for posttranslational modifications which include myristylation at Gly1, Asp-specific deamidation at Asn2 and autophosphorylation at Ser10. Previous reports have implicated specific biological features correlating with these modifications on Cα1. Since Cα2 is not modified in the same way as Cα1 we tested if they have distinct biochemical activities that may be reflected in different biological properties.

**Results:**

We show that Cα2 interacts with the two major forms of the regulatory subunit (R) of PKA, RI and RII, to form cAMP-sensitive PKAI and PKAII holoenzymes both *in vitro *and *in vivo *as is also the case with Cα1. Moreover, using Surface Plasmon Resonance (SPR), we show that the interaction patterns of the physiological inhibitors RI, RII and PKI were comparable for Cα2 and Cα1. This is also the case for their potency to inhibit catalytic activities of Cα2 and Cα1.

**Conclusion:**

We conclude that the regulatory complexes formed with either Cα1 or Cα2, respectively, are indistinguishable.

## Background

Cyclic 3', 5'-adenosine monophosphate (cAMP) is a key intracellular signaling molecule, whose main function is to activate the cAMP-dependent protein kinase (PKA) [1]. PKA is a heterotetrameric holoenzyme consisting of a regulatory (R) subunit dimer and two catalytic (C) subunits. The holoenzyme is activated when four molecules of cAMP bind to the R subunit dimer, two to each R subunit, releasing two free active C subunits [2;3]. In man, four different R subunits designated RIα, RIβ RIIα, RIIβ (reviewed in [4]), and four different C subunits (Cα, Cβ, Cγ and PrKX) have been identified [3]. The Cα and Cβ subunits are expressed in most tissues, while the Cγ subunit, which is transcribed from an intron-less gene, represents a retroposon derived from the Cα subunit [5]. Cγ is only expressed in human testis [6]. PrKX is an X chromosome-encoded protein kinase, and was identified as a PKA C subunit since it is inhibited by both PKI and RIα, and the RIα/PrKX complex is activated by cAMP [7].

Splice variants of both Cα and Cβ have been identified. In the case of Cα, two splice variants have been identified and termed Cα1 [8] and Cα2 [9-11]. Cα1 and Cα2 have non-identical N-terminal ends encoded by alternative use of two exons (1a and 1b, mouse terminology) located upstream of exon 2 in the murine Cα gene. The Cβ gene encodes a number of products identified in various species and have been designated Cβ1, Cβ2, Cβ3, Cβ4, Cβ3ab, Cβ3b, Cβ3abc, Cβ4ab, Cβ4b, Cβ4abc [12-17]. As is the case for the Cα forms, all the Cβ variants have variable N-terminal ends which are encoded by different exons upstream of exon 2 in the Cβ gene [14;15].

PKA-C splice variants are tissue-specifically expressed and some experimental evidence support that they may harbor specific features and non-identical activities when associated with the R subunits to form holoenzymes [18;19]. With regard to this Cα2 it is the sole C subunit expressed in the sperm cell. Moreover, Cα2 was shown to be vital for mouse sperm motility since ablation rendered the sperm cells non-motile and the male individuals infertile [9-11;20].

Cα1 is equipped with an N-terminal of 14 amino acids which undergo three well defined co- and posttranslational modifications. They include *in vivo *myristylation of Gly1 [21]. At position +1 an Asn is encoded which is partly deamidated *in vivo *leading to Cα1-Asp2 and Cα1-iso(β)Asp2 [22]. A third modification is identified as a PKA-autophosphorylation site at Ser10 [23-25]. Cα2 on the other hand is encoded with 7 unique amino acids at the N-terminus which to our knowledge do not have the ability to undergo any of the N-terminal modifications seen for Cα1.

Based on the different N-terminal sequences of Cα1 and Cα2 we speculate that they will introduce distinct biological features to these subunits. To investigate this hypothesis we made a thorough characterization of Cα2 activities both *in vivo *and *in vitro *and compared the results to what is known for Cα1 and to results obtained for Cα1 in the present work.

## Methods

### Sperm cell isolation

Semen samples were obtained from patients attending infertility investigations at the Andrology Laboratory at Rikshospitalet-Radiumhospitalet HF, Oslo, Norway. All patients signed a letter of approval and all experiments were done according to the recommendation from the Regional Committees for Medical and Health Research Ethics. All men produced their ejaculates on site or at home after 3-5 days of sexual abstinence. Samples were collected by masturbation into a wide-mouthed sterile container (Sarstedt Ltd., Leicester, United Kingdom) and after 30 min of liquefaction at 37°C, sperm parameters were evaluated according to World Health Organization (WHO) methods (World Health Organization, WHO laboratory manual for the examination of human semen and sperm-cervical mucus interaction (4th ed.), Cambridge University Press, Cambridge (1999).

Sperm cells were isolated from the seminal plasma by percoll gradient centrifugation. Sperm samples were pipetted on top of a 90%/45% percoll gradient and centrifuged at 2500 rpm for 20 min, no brake. After centrifugation, the sperm pellet was recovered by first using a sterile glass Pasteur pipette to remove the top layers of the semen sample and sperm gradient, leaving approximately 0.5 mL of the bottom layer. The sperm pellet was subsequently resuspended and washed twice in phosphate buffered saline (PBS) and centrifuged again at 2500 rpm for 8 min.

### Sperm head and tail separation

Isolated sperm cells were diluted to 1 mill/mL in PBS and sonicated mildly for 10 sec at low frequency. Ten μL samples were taken out to be examined by microscopy to assure head and tail separation. After complete separation the mixture was centrifuged at 400 g for 10 min. The supernatant containing the tails was transferred to a new tube and tails pelleted by centrifugation at 10.000 × g for 15 min. The tail pellet and the pellet from the first centrifugation were separately solubelized in RIPA buffer (10 mM Tris-HCl pH 7.5, 1 mM EDTA, 1% Triton X-100, 0.1% SDS, 0.1% Na-deoxycholate and 100 mM NaCl) containing 1 mM DTT, 1 mM PMSF and protease inhibitor cocktail (Roche), and sonicated 2 × 10 seconds at full effect.

### SDS-polyacrylamide gel electrophoresis (SDS-PAGE)

SDS-PAGE, was performed as described by [26]. Briefly, samples were diluted in SDS sample buffer (62.5 mM Tris-HCl, pH 6.8, 2.3% SDS, 10% glycerol, 5% β-mercaptoethanol, 0.001% bromophenol blue), boiled for 2 min and loaded onto slab gels consisting of a 4.5% stacking gel and a 12.5% separating gel.

### Immunoprecipitation

Lysates were cleared by centrifugation at 15000 g for 30 min at 4°C, and subsequently incubated with primary antibody [anti-Cα2 (SNO101; 320 μg/mL), mouse anti-RIα (2.5 μg/mL), rabbit anti-RIIα serum diluted 1:100] for 2 h to overnight. Antibody-antigen complexes were precipitated using either Dynabeads protein G (Dynal, catalogue number 100.04), anti-mouse agarose beads or anti-rabbit agarose beads (Sigma, catalogue number A6531, A1027). Precipitates were washed three times using appropriate buffer and extracted with buffer in the presence or absence of 1 mm 8-CPT cAMP as indicated in the figure legends.

### Immunoblot analysis

Total protein was estimated by Bradford protein assay (BioRad). Proteins were separated by SDS-PAGE and transferred to PVDF membranes by electro blotting. Membranes were blocked in 5% skimmed milk powder in Tris-buffered saline containing 0.1% Tween-20 (TBST) for 1 h at room temperature, and then incubated for 1 h at room temperature or overnight at 4°C with the appropriate primary antibodies diluted in TBST rabbit polyclonal anti-C 1:1000 (Santa Cruz Biotechnology), anti-Cα2 (SNO101), mouse anti-RIα 1:250 and rabbit anti-RIIα serum [27]. Membranes were washed for about 1 h in TBST and further incubated with HRP-conjugated secondary antibodies (ICN Diagnostics). Membranes were washed and finally developed using SuperSignal^© ^West Pico Chemiluminescent (Pierce).

### Endogenous sperm cell protein fractionation

Sperm cells were homogenized in PE buffer (5 mM KH_2_PO_4_, 5 mM K_2_HPO_4_, 1 mM EDTA, pH 6.8) containing 0.5% Triton X-100, 1 mM DTT, 1 mM PMSF and protease inhibitor cocktail (Roche), and sonicated 2 × 10 seconds. DEAE cellulose (Whatman DE 52) was applied to a column and equilibrated with PE buffer with 250 mM sucrose. The sperm cell homogenates were cleared by centrifugation at 15.000 g for 30 min at 4°C and applied to the column. After washing with PE buffer, the column was eluted with a linear 0-400 mM NaCl gradient created by a gradient mixer (total volume 50 ml), 1 ml fractions were collected and salt concentration checked by refractometry. Every other fraction, starting with fraction 1 was subjected to phosphotransferase activity measurements in the absence and presence of cAMP. Peak fractions were concentrated to 100 μl using centrifugal filters with 30 kDa cutoff (Millipore) and subjected to immunoblot analysis.

### Phosphotransferase assay

Catalytic activity of PKA was assayed by phosphorylating the PKA specific substrate Kemptide (Peninsula) using γ-[^32^P]ATP (5000 mCi/μmol) as previously described [26].

Specific activity is determined as U/mg, which is defined as μmol/mg × min.

### cAMP-binding assay

Determination of specific cAMP-binding of soluble R subunits was carried out in a buffer containing 0.3 μmol [^3^H]-cAMP (spec. act. 41.7 Ci/mmol, Amersham) [27].

### Activation Assay

The activation assay of Cα2 and myrCα1 with RIα and RIIβ, respectively, was performed in a spectrophotometric kinase activity assay as described earlier [28].

### Expression of Cα2, Cα1 and myristylated Cα1

Recombinant non-myristylated human Cα1 was expressed and purified as described previously [29;30]. Recombinant human Cα1, as well as recombinant human Ca2, were co-transformed with N-myristyl-transferase in Escherichia coli BL21 (DE3) (Novagen) and co-expressed with human Cα1 as well as Cα2 using the same conditions. Both proteins were purified by affinity chromatography using PKI-peptide Affi-Gel. The procedure was first described by Olsen *et al*. [31] and modified after Thullner *et al*. [32].

### Expression and purification of R subunits

Recombinant human R subunits (hRIα, hRIβ, hRIIα, hRIIβ) were over-expressed in Escherichia coli BL21 (DE3) RIL (Novagen) and purified according to a procedure by Bertinetti *et al*. [33] using Sp-8-AEA-cAMPS-agarose.

The purity of the R and C proteins was confirmed by SDS-PAGE as well as by immunoblot analysis and the biological activity of the proteins was measured as described before [30]. Primary sequence of Cα2 and the presence of N-myristylation at Cα1 were checked by mass spectrometry (Data not shown).

### SPR analysis

All SPR interaction analyses were performed at 25°C in 20 mM MOPS pH 7, 150 mM NaCl plus 0.005% (v/v) surfactant P20, 1 mM ATP, 5 mM MgCl_2 _and 50 μM EDTA using Biacore 2000 or 3000 instruments (GE Healthcare-Biacore, Sweden). For covalent coupling of the C subunits, carboxymethylated sensor chip surfaces (CM5, research grade, GE Healthcare) were activated with NHS/EDC for 7 min and non-myristylated Cα1, Cα2 and myrCα1 (5 μg/μl in 10 mM sodium acetate plus 200 μM ATP and 500 μM MgCl_2 _with a pH 6.0) as described [34] were injected on separate flow cells with a flow rate of 5 μl/min until approximately 300 response units (RU 1,000 RU = 1 ng/mm2 for a CM 5 chip) [35] were reached. This amine coupling was described previously [36;37]. Deactivation of the surface was performed using 1 M ethanolamine-HCl (pH 8.5) for 7 min. As a reference one flow cell was activated and deactivated in the absence of any protein.

The interaction experiments with four R subunit were performed at 25°C in running buffer (20 mM MOPS pH 7, 150 mM NaCl, 1 mM ATP, 5 mM MgCl_2_, 50 μM EDTA, 0.005% surfactant P20) at a flow rate of 30 μl/min. During simultaneous R subunit injection over the four surfaces (150 sec), the dissociation phase was monitored for 150 sec. The binding of R subunit to C subunit was not limited by mass transport according to previous experiments [38]. Response of the activated/deactivated reference cell was subtracted. Surfaces were regenerated with two subsequent 1 min injections of 0,1 mM cAMP, 2,5 mM EDTA in running buffer. Evaluation of non-normalized data was performed with Biaevaluation 3.2 RC1 (GE Healthcare). A Langmuir 1:1 binding model was applied for the kinetic analysis of C subunit/R subunit interactions [37].

The sensor chip was stored at 4°C in running buffer and tested for proper performance prior to each analysis.

## Results

### Sperm cell-specific Cα2 associates with RIα and RIIα in a cAMP-dependent fashion to form PKAI and PKAII holoenzymes in vivo

**Figure 1:**
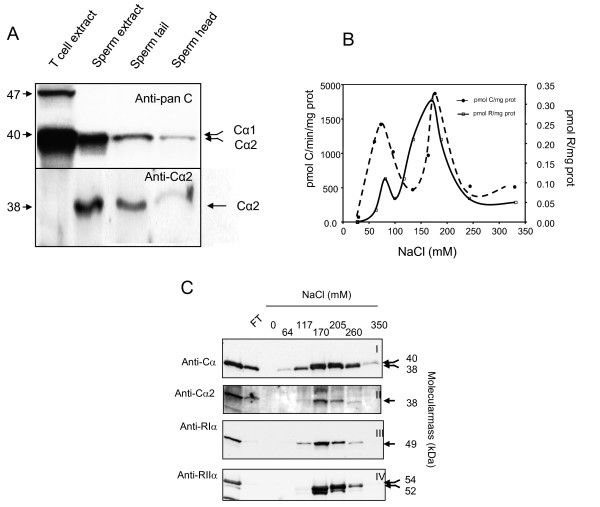
**Cα2 is expressed and associate with RIα and RIIα to form PKA type I and PKA type II in the whole sperm cell**. Panel A: Protein extracts of T cells (lane 1), total sperm (lane 2), sperm tail (lane 3) and sperm heads (lane 4) were separated by SDS-PAGE (12.5% gels) and transferred to PVDF filters for immunoblot analysis. The filters were incubated with a panC antiserum (anti-Cα (scbt), 1:1000) (upper panel) and an anti-Cα2 antiserum (SNO 101,1:20, see Methods) (lower panel). Arrows to the left indicates migration of the molecular weight markers. Arrows on the right indicate the identity of the C subunits. Panel B: PKA type I and II were eluted from DEAE-cellulose columns using increasing concentrations of NaCl (0-400 mM). The eluted fractions were analyzed for specific [3H]-cAMP-binding (dotted line open square) and C subunit- specific phosphotransferase activity (dotted line closed circle). Panel C: Protein fractions eluted from the DEAE columns were separated by SDS-PAGE (12.5% gels) and transferred to PVDF filters for immunoblot analysis. The filters were incubated with antibodies to the C (upper two panels) and R subunits (lower two panels). Immunoreactive proteins to anti-panC (anti-Cα, scbt, 1:1000) and anti-Cα2 (SNO 101, 1:20) elute between 64 and 260 mM NaCl. Immunoreactive proteins to anti-RIα (4D7, 1:200) elute between 64 and 260 mM NaCl and immunoreactive proteins to anti-RIIα (Crl. 1:500) between 170 and 260 mM NaCl. Arrows on the right indicates the relative molecular mass (kDa) for the respective C and R subunits.

**Figure 2:**
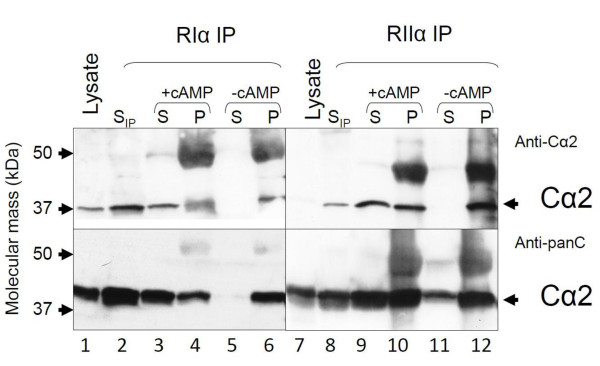
**Cα2 associate with both RIα and RIIα in a cAMP-dependent manner in sperm cells**. Sperm cell extracts were immunoprecipitated (IPed) with anti-RIα (1:100) and anti-RIIα (1:100). IPed proteins were separated by SDS-PAGE in 12. 5% gels, transferred to PVDF filters which were incubated with anti-Cα2 (1:20, upper panels) and a pan C antiserum (anti-Cγ (scbt) lower panels). Immunoreactive proteins which had been IPed (lanes 3-6 and lanes 9-12) were compared to immunoreactive proteins in the supernatant after the IP (lanes 2 and 8, S_IP_) and to total input (lanes 1 and 7, Lysates). Cell extracts IPed with anti-RIα and anti-RIIα and sepharose beads coated with anti-IgG, were centrifuged and the pellets washed with a buffer with (+cAMP) or without (-cAMP) cAMP. Washed pellets (P) and the respective supernatants (S) were analyzed by SDS-PAGE and immunoblotting. Lanes 2 and 8 show anti-Cα2 immunoreactive proteins left in the supernatants after anti-RIα and RIIα IP (S_IP_). Lanes 3 and 9 show anti-Cα2 immunoreactive proteins in supernatant of cAMP treated IP pellets. Lanes 4 and 10 show anti-Cα2 immunoreactive proteins in IP pellet after treatment with cAMP. Lanes 5 and 11 show anti-Cα2 immunoreactive proteins in supernatants of non-treated IP pellets. Lanes 6 and 12 show anti-Cα2 immunoreactive proteins in untreated IP pellet. Arrows on the left indicates migration of the molecular weight markers. Arrows on the right indicate the identity of the C subunit.

We first determined whether human sperm cells express Cα2. Protein extracts of T cells, whole sperm, sperm tail, and sperm heads were separated by SDS-PAGE transferred to immunoblot filters and filters probed with a pan-C antibody (Figure 1A, left panel, anti-C) and an anti-Cα2 antiserum (SNO 101, anti-Cα2 [10], Figure 1A, right panel). This confirms previous results that Cα2 is not expressed in T cells and is distributed in tail and head of spermatozoa [10]. RI and RII are both expressed in all sperm cell compartments and are known to form PKA type I and type II (PKAI and PKAII) [39-41]. PKAI and PKAII holoenzymes can be separated by DEAE ion exchange chromatography using increasing concentrations of NaCl [42]. Since Cα2 is the sole C subunit in human sperm cells it is expected that it will associate with RI and RII to form PKAI and PKAII, respectively, as has been demonstrated in the mouse [11]. To test this, whole sperm cell extracts (3 mg) were fractionated on DEAE resins by a linear NaCl gradient ranging from 0 to 350 mM). Two peaks of phosphotransferase- (--●--) and cAMP-binding activity (--□--) between 50-100 mM and 100-250 mM NaCl, respectively, were observed. This implied formation of both PKAI and PKAII (Figure 1B). C and R subunit identity were documented by immunoblotting using a pan-C antibody (Figure 1, panel CI) and anti-Cα2 (panel CII) as well as anti-RIα (panel CIII) and anti-RIIα (panel CIV). This showed that immunoreactive Cα2 co-elutes with the two major R subunits in sperm cells, RIα (peak I) and RIIα (peak II) [43-45]. From the figure it is also seen that some of the C activity was detected before the R subunit activity in the first peak, implying free C subunit. Moreover, we also noted that PKAI and PKAII containing Cα2 eluted at comparable concentrations of NaCl. To further investigate whether human Cα2 forms PKAI and PKAII in cell extracts we immunoprecipitated (IPed) with anti-RIα and anti-RIIα (Figure 2). Using immunoblotting and anti-Cα2 (upper panels) and anti- pan-C (lower panels) we showed that both RIα and RIIα associate with Cα2, implying that Cα2 forms PKAI and II *in vivo *(lanes 6 and 12). To define whether the R-C interaction is specific and functional, the IPed proteins were challenged with cAMP to dissociate the holoenzyme into R subunit dimers and free C subunits [46]. In these experiments the IPed R subunits would be expected to be immobilized by the precipitating antiserum and remain in the pellet (P) after the cAMP wash whereas the IPed C subunit would be released into the supernatant (S) in the presence of cAMP [46;47]. Figure 2 (lanes 3 and 4, and 9 and 10) depicts that immunoreactive Cα2 is released into the supernatant after the cAMP wash (+cAMP) after both anti-RIα and anti-RIIα IP (upper and lower panels) whereas the immunoreactive Cα2 subunit remained in the pellet (P) in the absence of cAMP (-cAMP) (Figure 2, lanes 5 and 6 and 11 and 12, upper and lower panels).

### Comparable activities of Cα2 and Cα1

**Figure 3:**
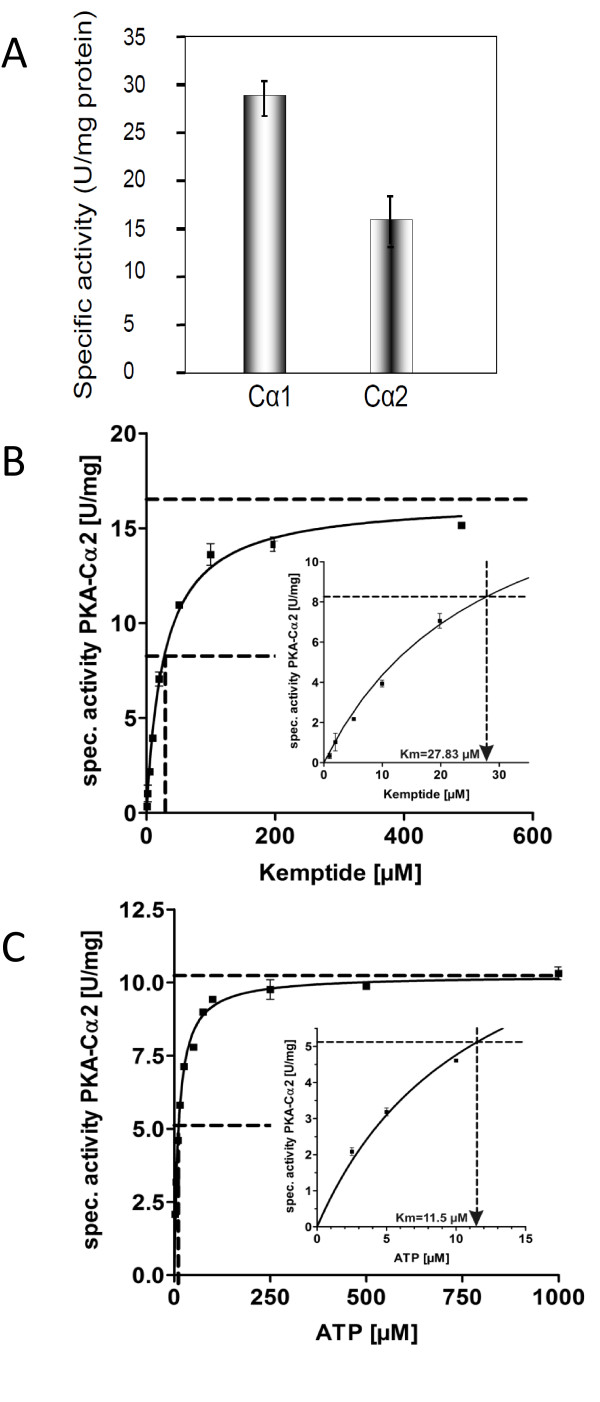
**Determination of specific activity, Km Kemptide and ATP for human Cα2**. Panel A: Specific activity of expressed Cα2 and Cα1 was determined to 18 ± 3 and 28 ± 4 units/mg, respectively. Note the lower activity of expressed Cα2 compared to Cα1. Km values for the PKA specific substrate Kemptide and ATP were determined by incubating recombinant Cα2 in the presence of increasing concentrations (1 to 500 μM) of the heptapeptide Kemptide (panel B) and ATP (panel C). Indirect spectrophotometric analyses of ATP consumption as measurement for Cα2 activity (see Methods) indicate a Km for Kemptide of 27.8 ± 2.3 μM (n = 2) and Km ATP of 11.5 ± 0.5 μM (n = 2), see insets panel B and C.

Our results and several reports showing that Cα2 associates with both RI and RII subunits [3; 48-50] in a cAMP-sensitive fashion imply that N-terminal differences do not interfere with PKA holoenzyme formation.. To further investigate wetherCα2 has activities that differ from Cα1 we expressed Cα2 using the pREST B vector. First we noted that Cα2 and Cα1 were captured in the soluble and particulate fractions of the bacteria lysates, respectively, suggesting differences in solubility (results not shown). Furthermore, specific activity of expressed Cα2 was determined to 18 ± 3 units/mg (U/mg, n = 3) which was notably lower than the specific activity of expressed Cα1 (28 ± 4 U/mg, Figure 3A). Taken together this may imply differential features of Cα1 and Cα2. Based on this and to determine the exact activities for Cα2 three features were investigated. These included (i) Cα2's substrate affinity, (ii) the ability to form holoenzymes with RI and RII *in vitro *and (iii) the Km values for Kemptide and ATP. In the latter case we found the Km values of Cα2 for Kemptide and ATP to be 27. 8 ± 2.3 μM and 11.5 ± 0.5 μM, respectively (Figure 3B and 3C). This is in good agreement with previous results obtained with expressed Cα1 [7].

**Figure 4:**
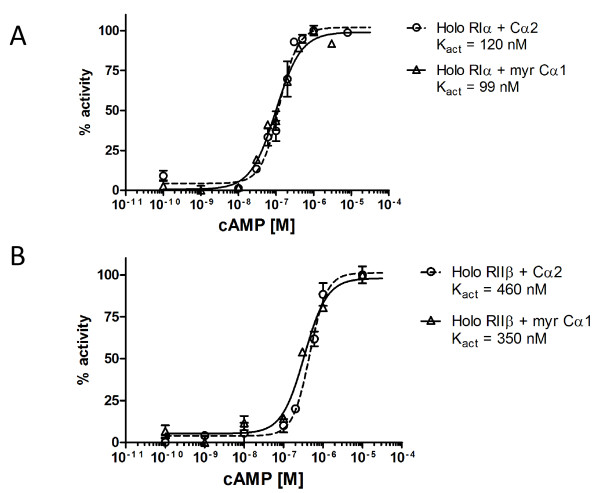
**Determination of the activation constant Kact (cAMP) for Cα2 and myr Cα1 containing PKA type I and II holoenzymes**. PKA type I and II holoenzymes were made by mixing recombinant Cα2 and myr Ca1, respectively, with either recombinant RIα (panel A) or RIIβ (panel B) in a molar ratio 1: 1.2 (C: R, see Methods). The holoenzymes were incubated with Kemptide (250 μM) and increasing concentrations of cAMP (0,1 nM- 10 μM) to determine the activation constant (Kact). A Kact (cAMP) for RIα-Cα2 and RIα-myrCα1. B Kact (cAMP) for RIIβ-Cα2 and RIIβ-myrCα1. .

We next monitored cAMP-sensitivity of type I and II PKA holoenzymes containing Cα2 *in vitro*. To calculate the accurate cAMP activation constant, PKAI (RIα) and PKAII (RIIβ) containing Cα2 holoenzymes were incubated with 250 μM Kemptide and 10 mM ATP in the presence of increasing concentrations of cAMP. Kact values for cAMP were 120 nM and 460 nM for the holoenzyme formed with RIα and RIIβ, respectively, showing that PKAI containing Cα2 are nearly 4 fold more sensitive to cAMP than PKAII containing Cα2 holoenzymes (figure 4A,B). The corresponding values for holoenzymes formed with myrCα1 were 99 nM (RIα) and 350 nM (RIIβ) (figure 4A,B) again demonstrating a 4 fold increased sensitivity for the RI holoenzyme. This is also in agreement with previous *in vitro *results for PKAI and II holoenzymes containing Cα1 [38;51], and the Kact values for mouse PKAI isolated from sperm cells ablated for PKAII (RIIα) [11]. It should be noted that Kact in wild type sperm cells which mainly express PKAII (RIIα-Cα2) [45] is almost identical to the Kact in RIIα ablated sperm cells. This may suggest that PKAI and PKAII display comparable Kact's *in vivo *and hence contradicts the *in vitro *results demonstrated previously [38] and by us here. It should also be noted that PKAI although expressed at low levels may skew the observed Kact values, due to its sensitivity for cAMP. To what extent this has biological consequences as has been demonstrated for PKAI and PKAII in lymphoid cells [27;52;53], remains to be tested.

**Figure 5:**
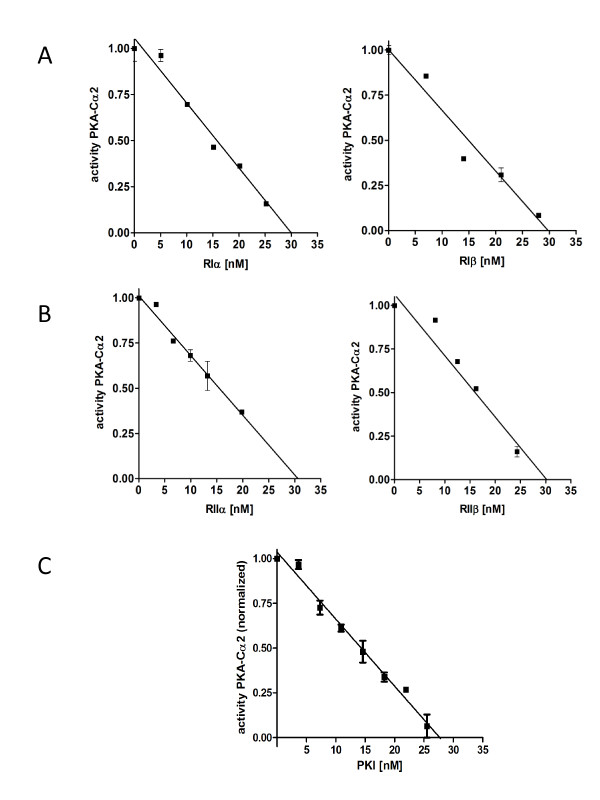
**Phosphotransferase activity of Cα2 is inhibited by the R subunits and PKI in dose-dependent manner**. Recombinant Cα2 (30 nM) mixed with Kemptide (250 μM) and ATP (10 mM) was incubated in the presence of increasing concentrations (0 - 25 nM) of either RIα and RIβ (panel A) and RIIα and RIIβ (panel B). Cα2 is inhibited by all R subunits in a dose-dependent manner at equimolar concentrations of RIα, RIβ, RIIα and RIIβ. Panel C: The inhibitory effect of PKIα was verified by incubating recombinant Cα2 (28 nM) in the presence of Kemptide (250 μM), ATP (10 mM) and increasing concentrations of recombinant PKIα (0-40 nM). Titration curves shown are normalized before linear regression (n = 2).

**Figure 6:**
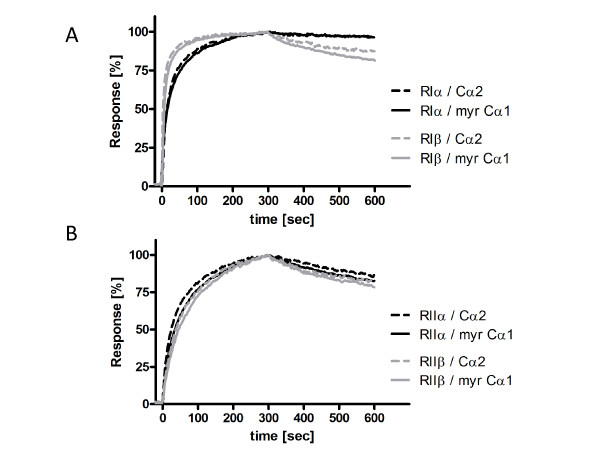
**The affinity of RI and RII subunits for Cα1 and Cα2 are comparable**. Cα2 and myrCα1 were immobilized on a CM5 sensor chip each with 300 RUs of on a surface using amine coupling (see Methods). Then recombinant RIα and RIβ (panel A) as well as RIIα and RIIβ (panel B) were run simultaneously over each Cα surface monitoring the association and dissociation of each R subunit at a flow rate of 30 μl/min. Association and dissociation were recorded for 5 min each and the interaction was measured with R subunits in concentrations ranging from 0.25 nM to 128 nM and 0.5 nM to 256 nM for type I and type II enzymes (for details see results), respectively (table 1). All experiments were performed in 20 mM MOPS pH 7, 150 mM NaCl plus 0.005% (v/v) surfactant P20, 1 mM ATP, 5 mM MgCl_2 _and 50 μM EDTA. The panels show representative kinetics injecting the four R-isoforms on myrCα1 and Cα2 as indicated on the normalized plot using 64 nM of each R subunit.

We then investigated the potency of the R subunits (RIα, RIIα, RIβ and RIIβ) and the protein kinase inhibitor PKIα to inhibit Cα2 phosphotransferase activity *in vitro*. Purified Cα2 (30 nM) was mixed with a fixed concentration of Kemptide (250 μM) in the presence of increasing concentrations of the various R subunits or PKIα. All the R subunits inhibited Cα2-dependent kinase activity by 50% at 15 nM and showed complete inhibition at a 1:1 molar ratio (Figure 5A and 5B). A fixed dose (28 nM) of Cα2 was inhibited by PKIα in a dose-dependent manner with complete inhibition at stoichiometric concentrations of Cα2 and PKIα (Figure 5C). The inhibitory effects of the various R subunits and PKI have previously been determined for Cα1 [7;29] and indicate that the efficiency in inhibiting Cα1 and Cα2 is similar for all R subunits and PKIα. Using a Biacore technology we next investigated the dissociation equilibrium constants (K_D_), association (k_ass_) and dissociation (k_diss_) rate constants for the various R subunits in association with either Cα1 or Cα2. We immobilized 300 RUs of myristylated Cα1 (myrCα1, see Methods) and Cα2 on separate flow cells of a CM5 Biacore sensor chip. Unmyristylated Cα1 was used as reference (data not shown). In the presence of 1 mM ATP and 5 mM MgCl_2 _the R subunits were simultaneously run over both C subunits on the sensor chip at a flow rate of 30 μL/min. In the case of RIα and RIβ they were run over the sensor chip at concentrations between 0.25 and 128 nM, and RIIα and RIIβ between 0.5 to 256 nM (raw data not shown). Figure 6 (panel A) shows representative runs of RIα, RIβ, RIIα and RIIβ (64 nM each; panel B) on Cα2 and myrCα1. The shape of the curves indicates that the relative on and off rates for RIα when associated with either Cα2 or myrCα1 were highly similar. The relative k_ass _values were slightly different, 1.6 × 10^6 ^and 1.9 × 10^6 ^M^-1^s^-1 ^for RIα versus myrCα1 and Cα2, respectively. The same was true for RIβ. However, in this case, although the KD value was almost identical to RIα, the association as well as the dissociation rate constants for RIβ was 2 times faster. Finally, no differences could be observed for the interaction of RIIα and RIIβ against myrCα1 versus Cα2 (for rate and equilibrium constants see Table 1).

**Table 1:** Association and dissociation constants of RI and RII and Cα1 and Cα2

Analyt/Ligand (immobilized)	**k**_**a **_**[M**^**-1**^**s**^**-1**^**]**	**k**_**d **_**[s]**	**K**_**D **_**[nM]**
hRIα/PKA-Cα1 myr	1.6E + 6	200,0E-6	0,13
hRIα/PKA-Cα2	1.9E + 6	214,0E-6	0,11

hRIβ/PKA-Cα1 myr	3.5E + 6	501,0E-6	0,15
hRIβ/PKA-Cα2	4.4E + 6	482,0E-6	0,11

hRIIα/PKA-Cα1 myr	1.0E + 6	469,0E-6	0,48
hRIIα/PKA-Cα2	1.2E + 6	433,0E-6	0,35

hRIIβ/PKA-Cα1 myr	0.5E + 6	797,0E-6	1,5
hRIIβ/PKA-Cα2	0.9E + 6	793,0E-6	0,9

**Figure 7:**
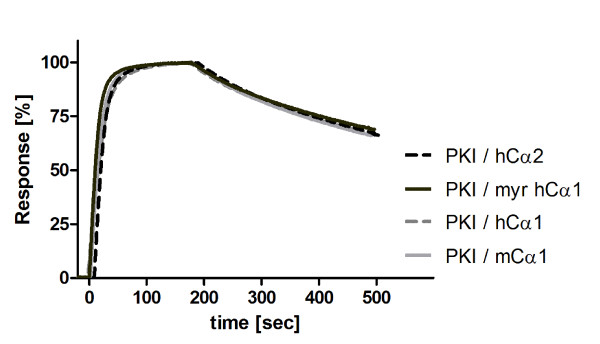
**PKIα binds almost identical to hCα1, myristylated hCα1, hCα2 and mCα1**. GST-PKIα was captured on an α-GST-Antibody sensor chip as described [55] to surface density of 300 RU. All C-subunits (Cα2, Cα1, myristylated Cα1 and, for comparison, mouse Cα1) were run over the GST-PKIα surface using the assay conditions as described in figure legend 6 in the presence of 1 mM ATP and 5 mM MgCl_2_. The plot shows the normalized binding data at a concentration of 25 nM C-subunit each.

In order to investigate the binding behavior of PKI, GST-PKIα was immobilized on sensor chips as described previously [54], and various concentrations of Cα2, Cα1, myrCα1 and, for comparison, mouse Cα1 were run at a flow rate of 30 μL/min over the sensor chips. This revealed a KD for all C subunits and PKIα at a range around 0,4 nM where the myrCα1 displayed a slightly faster association rate compared to Cα1 (4.9 × 10^6 ^and 3.2 × 10^6 ^M^-1^s^-1^, respectively) with all the dissociation rates being similar (1.5 × 10^-3 ^s^-1^) (Table 2). SPR measurements with single concentrations demonstrated almost identical shapes of the curves (Figure 7), indicating comparable association and dissociation rates. In order to determine accurate association rate constants, different concentrations of the respective C subunits were applied (results not shown), leading again to the conclusion that the binding activities of Cα2 and Cα1 for PKIα are highly similar.

**Table 2:** Association and dissociation constants of GST-PKIa for Ca1 and Cα2

Analyt/Ligand (immobilized)	**K**_**a**_**[M**^**-1**^**S**^**-1**^**]**	**K**_**d**_**[S]**	**K**_**d**_**[nM]**
GST-PKIα/PKA-Cα1	4.9 × 10^5^	1.5 × 10^-3^	0.5

GST-PKIα/PKA-Cα2	3.2 × 10^5^	1.5 × 10^-3^	0.7

## Discussion

At the protein level Cα1 and Cα2 are 97% homologous and only differ at the N-terminal end. Based on this we investigated to what extent differences at the N-terminus may influence splice variant-specific activities that may have biological importance. We found that Cα2 expressed in bacteria was not captured by inclusion bodies as was the case with Cα1. Moreover, the specific activity of Cα2 was lower compared to Cα1. Apart from these differences we observed that Cα2 was highly similar to Cα1 in all parameters measured. This included association of Cα2 with RI and RII to form cAMP-sensitive holoenzymes both *in vivo *and *in vitro*. Furthermore, Km values of Cα2 for Kemptide and ATP were comparable to those determined for Cα1. This was also the case for the ability of RI, RII and PKI to inhibit Cα2 enzyme activity *in vitro *(data not shown). Finally, K_D _values as measured by SPR were shown to be comparable between Cα1 and Cα2 towards the RI and RII subunits as well as PKI.

Several reports imply that N-terminal modifications of Cα1 introduce specific features that may have biological consequences. To this end it has been suggested that phosphorylation of Ser10 in Cα1 introduces an electrostatic force which may help the C subunit to remain soluble even when myristylated [55;56]. Moreover, two reports have demonstrated that the N-terminal myristyl moiety of Cα1 is embedded in a hydrophobic pocket encompassed in the large lobe [57;58]. Mutation of Gly1 to Ala rendering the Cα1 non-myristylated, demonstrated that myristylation was non-essential for conformation and enzyme activation, and was not required for Cα1 interaction with other proteins including various substrates and the R subunits [21,59]. The fact that Cα2 is not myristylated and displays comparable activities with myristylated Cα1 suggests that myristylation is not essential for catalytic activity, holoenzyme formation and inhibition by PKI. This is further supported in that deletion of the entire Cα1 N-terminus did not severely interfere with catalytic activity and inhibitor binding despite that deletion caused thermo instability [25]. This also suggests that the amino acids 2 (Asn) and 10 (Ser) of Cα1 are not essential for activity a suggestion which is supported by our results on Cα2.

Cα1 and Cβ1 which are 100% identical at the N-terminus, but only 91% identical in the sequence encode by exon 2 through 10, have different apparent sizes (40 and 41 kDa) and possess distinct biochemical properties both *in vitro *and *in vivo *[60; 31]. These differences include differential Km values for certain peptide substrates and that Cα1 but not Cβ1 is inhibited by substrate concentrations above 100 μM. In addition, they display distinct IC_50 _values for PKI and RIIα. Taken together with our results this may suggest that the amino acid sequence encoded by exon 2 through 10 and not exon 1 influence C subunit activities such as holoenzyme formation, enzyme activity and inhibition by R and PKI. This may further imply that Cα1 and Cβ1 have distinct roles in regulating cellular processes. This was recently shown in T cells which express Cα1, Cβ1 and Cβ2. In that study Cα1, but none of the Cβ forms, mediated the inhibitory effect of cAMP on immune cell reactivity *in vivo *[17;61]. In light of these observations it is also of interest to note that Cα2 but not Cα1 is required for sperm cell forward velocity and male fertility, despite 100% identity at the amino acid sequence encoded by exon 2 through 10 [20;62]. However, since Cα2 is the sole C subunit in sperm cells [9;10;63], the difference observed may only be ascribed to tissue-specific expression and not sequence-specific differences.

In contrast to Cα1 it is expected that the hydrophobic pocket in which the myristyl group is submersed in Cα1, is constitutively empty and exposed to the surroundings at all time in non-myristylated Cα2. It has been speculated whether exposure of the hydrophobic pocket would introduce more lipophilic properties to the Cα2 subunit [64]. Support for such a hypothesis is found in a by a study demonstrating that binding of Cα1 to RII induced a unique conformation that is associated with exposure of the hydrophobic pocket to the surroundings due to increase in N-terminal flexibility of the N-myristate away from the large lobe. This renders Cα1 more hydrophobic and promotes membrane association of the PKA II holoenzyme only [64]. Therefore it may be suggested that exposure of the hydrophobic pocket serves features such as isoform specific features and subcellular localization of the C subunit. To this end it is interesting to note that Cα2 is associated with the sperm tail in the presence of detergent treatment with 1% Triton X-100 .and, after a challenge with 2 mM cAMP [10]. This may be indicative of a direct association of Cα2 with subcellular structures. To what extent such attachment involves the hydrophobic pocket remains unknown. In other cells and tissues, C subunits targeted to subcellular structures independent of the R subunit and traditional A-kinase anchoring proteins have been demonstrated. To day a number of C subunit binding proteins have been identified. These include PKI, A-kinase interacting protein 1 (AKIP1), homologous to AKAP95 (HA95), inhibitor of NFkappaB kinase (IκB), Caveolin-1 and p75 neutrophine receptor (p75NTR) [65-69]. To what extent Cα2 is targeted to the sperm cell midpiece through a C interaction protein and if specificity of binding is retained in the hyper variable N-terminal end remains to be shown. However, it should be noted that deamination of the Asn2 moiety in Cα1 helps fine-tuning enzyme distribution within the cell *in vivo *[70]. Moreover, p75NTR was shown to specifically bind to the Cβ splice variant Cβ4ab [69], which is encoded with unique N-terminal domain that may not undergo the same posttranslational modifications as Cα1 [15;69]. Together this may imply that the N-terminal end may be important for targeting and specificity of subcellular localization of the various C subunits.

## Conclusion

Our study demonstrates that N-terminal sequence encoded by alternative use of exons upstream of exon 2 in the PRKACA gene does not influence C subunit activities such as holoenzyme formation, cAMP sensitivity, enzyme activity as well as inhibition by RI, RII and PKI. Based on several studies it may be suggested that the N-terminus is involved in other C subunit features such as subcellular localization.

## Authors' contributions

MMV has written the manuscript and has designed an contributed to the experiments in figures 3 through 7 and table 1 and 2. HMZ has contributed to the BIACORE experiments. EM has performed the experiments in figures 1 and 2. HVB has made recombinant proteins for Cα1 and Cα2 and contributed to the experiments in figure 3. FWH and BSS have conceived the ideas to the manuscript and contributed to the writing. All authors read and approved the final manuscript.

## Abbreviations

PKA: Protein kinase A; C: Catalytic subunit; Cα: Alpha-form of C; Cα2: Sperm-specific C subunit.

## References

[B1] Butcher RW Ho RJ Meng HC Sutherland EW Adenosine 3',5'-monophosphate in biological materials. II. The measurement of adenosine 3',5'-monophosphate in tissues and the role of the cyclic nucleotide in the lipolytic response of fat to epinephrine J Biol Chem 1965 240 4515 4523 4378937

[B2] Beebe SJ Corbin JD Krebs, E. G. & Boyer, P. D Cyclic nucleotide-dependent protein kinases 1986 Academic Press, Orlando London 43 111 17A

[B3] Skålhegg BS Tasken K Specificity in the cAMP/PKA signaling pathway. Differential expression, regulation, and subcellular localization of subunits of PKA Front Biosci 2000 5 D678 D693 10.2741/Skalhegg 10922298

[B4] Beebe SJ The cAMP-dependent protein kinases and cAMP signal transduction Semin Cancer Biol 1994 5 285 294 7803765

[B5] Reinton N Haugen TB Ørstavik S Skålhegg BS Hansson V Jahnsen T Tasken K The gene encoding the C gamma catalytic subunit of cAMP-dependent protein kinase is a transcribed retroposon Genomics 1998 49 290 297 10.1006/geno.1998.5240 9598317

[B6] Beebe SJ Øyen O Sandberg M Frøysa A Hansson V Jahnsen T Molecular cloning of a tissue-specific protein kinase (C gamma) from human testis--representing a third isoform for the catalytic subunit of cAMP-dependent protein kinase Mol Endocrinol 1990 4 465 475 10.1210/mend-4-3-465 2342480

[B7] Zimmermann B Chiorini JA Ma Y Kotin RM Herberg FW PrKX is a novel catalytic subunit of the cAMP-dependent protein kinase regulated by the regulatory subunit type I J Biol Chem 1999 274 5370 5378 10.1074/jbc.274.9.5370 10026146

[B8] Showers MO Maurer RA Cloning of cDNA for the catalytic subunit of cAMP-dependent protein kinase Methods Enzymol 1988 159 311 318 3412179 10.1016/0076-6879(88)59031-6

[B9] San Agustin JT Witman GB Differential expression of the C(s) and Calpha1 isoforms of the catalytic subunit of cyclic 3',5'-adenosine monophosphate-dependent protein kinase testicular cells Biol Reprod 2001 65 151 164 10.1095/biolreprod65.1.151 11420235

[B10] Reinton N Ørstavik S Haugen TB Jahnsen T Tasken K Skålhegg BS A novel isoform of human cyclic 3',5'-adenosine monophosphate-dependent protein kinase, c alpha-s, localizes to sperm midpiece Biol Reprod 2000 63 607 611 10.1095/biolreprod63.2.607 10906071

[B11] Desseyn JL Burton KA McKnight GS Expression of a nonmyristylated variant of the catalytic subunit of protein kinase A during male germ-cell development Proc Natl Acad Sci USA 2000 97 6433 6438 10.1073/pnas.97.12.6433 10841548 PMC18620

[B12] Uhler MD Chrivia JC McKnight GS Evidence for a second isoform of the catalytic subunit of cAMP-dependent protein kinase J Biol Chem 1986 261 15360 15363 3023318

[B13] Wiemann S Kinzel V Pyerin W Isoform C beta 2, an unusual form of the bovine catalytic subunit of cAMP-dependent protein kinase J Biol Chem 1991 266 5140 5146 2002051

[B14] Guthrie CR Skålhegg BS McKnight GS Two novel brain-specific splice variants of the murine Cbeta gene of cAMP-dependent protein kinase J Biol Chem 1997 272 29560 29565 10.1074/jbc.272.47.29560 9368018

[B15] Ørstavik S Reinton N Frengen E Langeland BT Jahnsen T Skålhegg BS Identification of novel splice variants of the human catalytic subunit Cbeta of cAMP-dependent protein kinas Eur J Biochem 2001 268 5066 5073 10.1046/j.0014-2956.2001.02429.x 11589697

[B16] Kvissel AK Ørstavik S Øistad P Rootwelt T Jahnsen T Skålhegg BS Induction of Cbeta splice variants and formation of novel forms of protein kinase A type II holoenzymes during retinoic acid-induced differentiation of human NT2 cells Cell Signal 2004 16 577 587 10.1016/j.cellsig.2003.08.014 14751543

[B17] Funderud A Henanger HH Hafte TT Amieux PS Ørstavik S Skålhegg BS Identification, cloning and characterization of a novel 47 kDa murine PKA C subunit homologous to human and bovine Cbeta2 BMC Biochem 2006 7 20 10.1186/1471-2091-7-20 16889664 PMC1557514

[B18] Qi M Zhuo M Skålhegg BS Brandon EP Kandel ER McKnight GS Idzerda RL Impaired hippocampal plasticity in mice lacking the Cbeta1 catalytic subunit of cAMP-dependent protein kinase Proc Natl Acad Sci USA 1996 93 1571 1576 10.1073/pnas.93.4.1571 8643673 PMC39982

[B19] Gamm DM Baude EJ Uhler MD The major catalytic subunit isoforms of cAMP-dependent protein kinase have distinct biochemical properties in vitro and in vivo J Biol Chem 1996 271 15736 15742 10.1074/jbc.271.26.15736 8662989

[B20] Skålhegg BS Huang Y Su T Idzerda RL McKnight GS Burton KA Mutation of the Calpha subunit of PKA leads to growth retardation and sperm dysfunction Mol Endocrinol 2002 16 630 639 10.1210/me.16.3.630 11875122

[B21] Clegg CH Ran W Uhler MD McKnight GS A mutation in the catalytic subunit of protein kinase A prevents myristylation but does not inhibit biological activity J Biol Chem 1989 264 20140 20146 2584209

[B22] Jedrzejewski PT Girod A Tholey A Konig N Thullner S Kinzel V Bossemeyer D A conserved deamidation site at Asn 2 in the catalytic subunit of mammalian cAMP-dependent protein kinase detected by capillary LC-MS and tandem mass spectrometry Protein Sci 87 457 469 199 10.1002/pro.5560070227 PMC2143929 9521123

[B23] Toner-Webb J van Patten SM Walsh DA Taylor SS Autophosphorylation of the catalytic subunit of cAMP-dependent protein kinase J Biol Chem 1992 267 25174 25180 1460017

[B24] Yonemoto W McGlone ML Grant B Taylor SS Autophosphorylation of the catalytic subunit of cAMP-dependent protein kinase in Escherichia coli Protein Eng 1997 10 915 925 10.1093/protein/10.8.915 9415441

[B25] Herberg FW Zimmermann B McGlone M Taylor SS Importance of the A-helix of the catalytic subunit of cAMP-dependent protein kinase for stability and for orienting subdomains at the cleft interface Protein Sci 1997 6 569 579 9070439 10.1002/pro.5560060306 PMC2143671

[B26] Kvissel AK Ørstavik S Eikvar S Brede G Jahnsen T Collas P Akusjarvi G Skålhegg BS Involvement of the catalytic subunit of protein kinase A and of HA95 in pre-mRNA splicing Exp Cell Res 2007 313 2795 2809 10.1016/j.yexcr.2007.05.014 17594903

[B27] Skålhegg BS Landmark BF Doskeland SO Hansson V Lea T Jahnsen T Cyclic AMP-dependent protein kinase type I mediates the inhibitory effects of 3',5'-cyclic adenosine monophosphate on cell replication in human T lymphocytes J Biol Chem 1992 267 15707 15714 1379235

[B28] Diskar M Zenn HM Kaupisch A Prinz A Herberg FW Molecular basis for isoform-specific autoregulation of protein kinase A Cellular Signalling 2007 19 2024 2034 10.1016/j.cellsig.2007.05.012 17614255

[B29] Herberg FW Taylor SS Physiological inhibitors of the catalytic subunit of cAMP-dependent protein kinase: effect of MgATP on protein-protein interactions Biochemistry 1993 32 14015 14022 10.1021/bi00213a035 8268180

[B30] Slice LW Taylor SS Expression of the catalytic subunit of cAMP-dependent protein kinase in Escherichia coli J Biol Chem 1989 264 20940 20946 2687267

[B31] Olsen SR Uhler MD Affinity purification of the C alpha and C beta isoforms of the catalytic subunit of cAMP-dependent protein kinase J Biol Chem 1989 264 18662 18666 2553718

[B32] Thullner S Gesellchen F Wiemann S Pyerin W Kinzel V Bossemeyer D The protein kinase A catalytic subunit Cbeta2: molecular characterization and distribution of the splice variant Biochem J 2000 351 123 132 10.1042/0264-6021:3510123 10998354 PMC1221342

[B33] Bertinetti D Schweinsberg S Hanke SE Schwede F Bertinetti O Drewianka S Genieser HG Herberg FW Chemical tools selectively target components of the PKA system BMC Chem Biol 2009 9 3 10.1186/1472-6769-9-3 19216744 PMC2660902

[B34] Gesellchen F Zimmermann B Herberg FW Direct optical detection of protein-ligand interactions Methods Mol Biol 2005 305 17 46 15939992 10.1385/1-59259-912-5:017

[B35] Löfås S Malmqvist M Rönnberg I Stenberg E Liedberg B Lundström I Quantitative determination of surface concentration of protein with surface plasmon resonance using radio labeled proteins Sensors and Actuators B: Chemical 1991 5 79 84 10.1016/0925-4005(91)80224-8

[B36] Herberg FW Dostmann WR Zorn M Davis S Taylor SS Crosstalk between domains in the regulatory subunit of cAMP-dependent protein kinase: influence of amino terminus on cAMP binding and holoenzyme formation Biochemistry 1994 33 7485 7494 10.1021/bi00189a057 8003514

[B37] Hahnefeld C Drewianka S Herberg FW Determination of kinetic data using surface plasmon resonance biosensors Methods Mol Med 2004 94 299 320 14959837 10.1385/1-59259-679-7:299

[B38] Herberg FW Doyle ML Cox S Taylor SS Dissection of the nucleotide and metal-phosphate binding sites in cAMP-dependent protein kinase Biochemistry 1999 38 6352 6360 10.1021/bi982672w 10320366

[B39] Pariset C Feinberg J Dacheux JL Øyen O Jahnsen T Weinman S Differential expression and subcellular localization for subunits of cAMP-dependent protein kinase during ram spermatogenesis J Cell Biol 1989 109 1195 1205 10.1083/jcb.109.3.1195 2768339 PMC2115753

[B40] Pariset C Weinman S Differential localization of two isoforms of the regulatory subunit RII alpha of cAMP-dependent protein kinase in human sperm: biochemical and cytochemical study Mol Reprod Dev 1994 39 415 422 10.1002/mrd.1080390410 7893490

[B41] Øye O Myklebust F Scott JD Cadd GG McKnight GS Hansson V Jahnsen T Subunits of cyclic adenosine 3',5'-monophosphate-dependent protein kinase show differential and distinct expression patterns during germ cell differentiation: alternative polyadenylation in germ cells gives rise to unique smaller-sized mRNA species Biol Reprod 1990 43 46 54 10.1095/biolreprod43.1.46 2393692

[B42] Corbin JD Sugden PH West L Flockhart DA Lincoln TM McCarthy D Studies on the properties and mode of action of the purified regulatory subunit of bovine heart adenosine 3':5'-monophosphate-dependent protein kinase J Biol Chem 1978 253 3997 4003 206557

[B43] Cook PF Neville ME Jr Vrana KE Hartl FT Roskoski R Jr Adenosine cyclic 3',5'-monophosphate dependent protein kinase: kinetic mechanism for the bovine skeletal muscle catalytic subunit Biochemistry 1982 21 5794 5799 10.1021/bi00266a011 6295440

[B44] Landmark BF Fauske B Eskild W Skålhegg B Lohmann SM Hansson V Jahnsen T Beebe SJ Identification, characterization, and hormonal regulation of 3', 5'-cyclic adenosine monophosphate-dependent protein kinases in rat Sertoli cells Endocrinology 1991 129 2345 2354 10.1210/endo-129-5-2345 1657573

[B45] Landmark BF Øyen O Skålhegg BS Fauske B Jahnsen T Hansson V Cellular location and age-dependent changes of the regulatory subunits of cAMP-dependent protein kinase in rat testis J Reprod Fertil 1993 99 323 334 10.1530/jrf.0.0990323 8107013

[B46] Ørstavik S Funderud A Hafte TT Eikvar S Jahnsen T Skålhegg BS Identification and characterization of novel PKA holoenzymes in human T lymphocytes FEBS J 2005 272 1559 1567 10.1111/j.1742-4658.2005.04568.x 15794744

[B47] Larsen AC Kvissel AK Hafte TT Avellan CI Eikvar S Rootwelt T Ørstavik S Skålhegg BS Inactive forms of the catalytic subunit of protein kinase A are expressed in the brain of higher primates FEBS J 2008 275 250 262 18070107 10.1111/j.1742-4658.2007.06195.x

[B48] Burton KA Treash-Osio B Muller CH Dunphy EL McKnight GS Deletion of type IIalpha regulatory subunit delocalizes protein kinase A in mouse sperm without affecting motility or fertilization J Biol Chem 1999 274 24131 24136 10.1074/jbc.274.34.24131 10446185

[B49] Amieux PS Cummings DE Motamed K Brandon EP Wailes LA Le K Idzerda RL McKnight GS Compensatory regulation of RIalpha protein levels in protein kinase A mutant mice J Biol Chem 1997 272 3993 3998 10.1074/jbc.272.7.3993 9020105

[B50] Amieux PS McKnight GS The essential role of RI alpha in the maintenance of regulated PKA activity Ann N Y Acad Sci 2002 968 75 95 10.1111/j.1749-6632.2002.tb04328.x 12119269

[B51] Gibson RM Taylor SS Dissecting the cooperative reassociation of the regulatory and catalytic subunits of cAMP-dependent protein kinase. Role of Trp-196 in the catalytic subunit J Biol Chem 1997 272 31998 2005 10.1074/jbc.272.51.31998 9405392

[B52] Levy FO Rasmussen AM Tasken K Skålhegg BS Huitfeldt HS Funderud S Smeland EB Hansson V Cyclic AMP-dependent protein kinase (cAK) in human B cells: co-localization of type I cAK (RI alpha 2 C2) with the antigen receptor during anti-immunoglobulin-induced B cell activation Eur J Immunol 1996 26 1290 1296 10.1002/eji.1830260617 8647207

[B53] Torgersen KM Vaage JT Levy FO Hansson V Rolstad B Tasken K Selective activation of cAMP-dependent protein kinase type I inhibits rat natural killer cell cytotoxicity J Biol Chem 1997 272 5495 5500 10.1074/jbc.272.9.5495 9038153

[B54] Zimmermann B Schweinsberg S Drewianka S Herberg FW Effect of metal ions on high-affinity binding of pseudosubstrate inhibitors to PKA Biochem J 2008 413 93 101 10.1042/BJ20071665 18373497

[B55] McLaughlin S Aderem A The myristoyl-electrostatic switch: a modulator of reversible protein-membrane interactions Trends Biochem Sci 1995 20 272 276 10.1016/S0968-0004(00)89042-8 7667880

[B56] Hanakam F Albrecht R Eckerskorn C Matzner M Gerisch G Myristoylated and non-myristoylated forms of the pH sensor protein hisactophilin II: intracellular shuttling to plasma membrane and nucleus monitored in real time by a fusion with green fluorescent protein EMBO J 1996 15 2935 2943 8670794 PMC450234

[B57] Bossemeyer D Engh RA Kinzel V Ponstingl H Huber R Phosphotransferase and substrate binding mechanism of the cAMP-dependent protein kinase catalytic subunit from porcine heart as deduced from the 2.0 A structure of the complex with Mn2+ adenylyl imidodiphosphate and inhibitor peptide PKI(5-24) EMBO J 1993 12 849 859 8384554 10.1002/j.1460-2075.1993.tb05725.x PMC413283

[B58] Zheng JH Knighton DR Parello J Taylor SS Sowadski JM Crystallization of catalytic subunit of adenosine cyclic monophosphate-dependent protein kinase Methods Enzymol 1991 200 508 521 1956335 10.1016/0076-6879(91)00167-u

[B59] Clegg CH Correll LA Cadd GG McKnight GS Inhibition of intracellular cAMP-dependent protein kinase using mutant genes of the regulatory type I subunit J Biol Chem 1987 262 13111 13119 2820963

[B60] Uhler MD Carmichael DF Lee DC Chrivia JC Krebs EG McKnight GS Isolation of cDNA clones coding for the catalytic subunit of mouse cAMP-dependent protein kinase Proc Natl Acad Sci USA 1986 83 1300 1304 10.1073/pnas.83.5.1300 3456589 PMC323063

[B61] Funderud A Ås-Hanssen K Aksaas AK Hafte TT Corthay A Munthe LA Ørstavik S Skålhegg BS Isoform-specific regulation of immune cell reactivity by the catalytic subunit of protein kinase A (PKA) Cell Signal 2009 21 274 281 10.1016/j.cellsig.2008.10.013 19000925

[B62] Nolan MA Babcock DF Wennemuth G Brown W Burton KA McKnight GS Sperm-specific protein kinase A catalytic subunit Calpha2 orchestrates cAMP signaling for male fertility Proc Natl Acad Sci USA 2004 101 13483 13488 10.1073/pnas.0405580101 15340140 PMC518783

[B63] San Agustin JT Leszyk JD Nuwaysir LM Witman GB The catalytic subunit of the cAMP-dependent protein kinase of ovine sperm flagella has a unique amino-terminal sequence J Biol Chem 1998 273 24874 24883 10.1074/jbc.273.38.24874 9733793

[B64] Gangal M Clifford T Deich J Cheng X Taylor SS Johnson DA Mobilization of the A-kinase N-myristate through an isoform-specific intermolecular switch Proc Natl Acad Sci USA 1999 96 12394 12399 10.1073/pnas.96.22.12394 10535933 PMC22929

[B65] Sastri M Barraclough DM Carmichael PT Taylor SS A-kinase-interacting protein localizes protein kinase A in the nucleus Proc Natl Acad Sci USA 2005 102 349 354 10.1073/pnas.0408608102 15630084 PMC544310

[B66] Han I Xue Y Harada S Ørstavik S Skålhegg B Kieff E Protein kinase A associates with HA95 and affects transcriptional coactivation by Epstein-Barr virus nuclear proteins Mol Cell Biol 2002 22 2136 2146 10.1128/MCB.22.7.2136-2146.2002 11884601 PMC133669

[B67] Zhong H SuYang H Erdjument-Bromage H Tempst P Ghosh S The transcriptional activity of NF-kappaB is regulated by the IkappaB-associated PKAc subunit through a cyclic AMP-independent mechanism Cell 1997 89 413 424 10.1016/S0092-8674(00)80222-6 9150141

[B68] Razani B Lisanti MP Two distinct caveolin-1 domains mediate the functional interaction of caveolin-1 with protein kinase A Am J Physiol Cell Physiol 2001 281 C1241 C1250 11546661 10.1152/ajpcell.2001.281.4.C1241

[B69] Higuchi H Yamashita T Yoshikawa H Tohyama M PKA phosphorylates the p75 receptor and regulates its localization to lipid rafts EMBO J 2003 22 1790 1800 10.1093/emboj/cdg177 12682012 PMC154469

[B70] Pepperkok R Hotz-Wagenblatt A Konig N Girod A Bossemeyer D Kinzel V Intracellular distribution of mammalian protein kinase A catalytic subunit altered by conserved Asn2 deamidation J Cell Biol 2000 148 715 726 10.1083/jcb.148.4.715 10684253 PMC2169370

